# Personal Protective Equipment and Headaches: Cross-Sectional Study Among Moroccan Healthcare Workers During COVID-19 Pandemic

**DOI:** 10.7759/cureus.12047

**Published:** 2020-12-13

**Authors:** Amal Hajjij, Jehanne Aasfara, Mohamed Khalis, Hamid Ouhabi, Fouad Benariba, Chafik El Kettani

**Affiliations:** 1 Department of Otolaryngology, Head and Neck Surgery, Cheikh Khalifa International University Hospital, Faculty of Medicine, Mohammed VI University of Health Sciences (UM6SS), Casablanca, MAR; 2 Department of Neurology, Cheikh Khalifa International University Hospital, Faculty of Medicine, Mohammed VI University of Health Sciences (UM6SS), Casablanca, MAR; 3 Department of Epidemiology and Public Health, Mohammed VI University of Health Sciences (UM6SS), Casablanca, MAR; 4 Department of Otolaryngology, Head and Neck Surgery, Mohammed V Military Training Hospital, Rabat, MAR; 5 Department of Anesthesiology and Reanimation, Cheikh Khalifa International University Hospital, Faculty of Medicine, Mohammed VI University of Health Sciences (UM6SS), Casablanca, MAR

**Keywords:** headache, covid-19, personal protective equipment, healthcare workers, n95 masks, eye protection, hospital

## Abstract

Background

Healthcare workers in frontline during the coronavirus 19 disease (COVID-19) pandemic are mandated to wear specific personal protective equipment (PPE) including high filtrating masks and/or eye protection during extended period of time. Compressive headaches secondary to PPE use including N95 masks have been reported. We aim to describe subtypes of headache related to PPE use in our hospital in Casablanca and working condition factors associated with it.

Methods

We conducted a cross-sectional study among healthcare workers in frontline at Cheikh khalifa International University Hospital, using an online questionnaire. We collected demographic data, comorbidities and previous headaches history. Data about working conditions during pandemic, type and duration of PPE use were described. We calculated the prevalence of De Novo or an aggravated headache among healthcare workers. We studied correlations between PPE related headaches and working conditions and trends in PPE use during the pandemic. Finally, we described the overall discomfort related to PPE use.

Results

A total of 155 healthcare workers responded to the questionnaire. The N95 masks were the most used type (95.5%) associated with an eye protection in 61.3%. The overall prevalence of headache related to PPE was 62%. It was experienced De Novo in 32.9%, while it was an aggravation of pre-existing headache in 29%. Working more than 8 hours per shift during the pandemic was correlated to De novo headache (p = 0.008). The profession of doctor and working more than 12 hours per shift were correlated to aggravated headache (p = 0.02, p = 0.023). Healthcare workers experienced moderate discomfort, blurred vision and reduced concentration. They judged their professional performance mildly reduced by the use of PPE.

Conclusion

The increased use of PPE, especially high filtrating masks during the COVID-19 outbreak is responsible for generating headaches in healthcare workers on frontline either De novo or as an aggravation of pre-existing one. Working conditions have the greater impact on generating these types of headaches more than any pre-existing comorbidity. These findings should be considered to accommodate health care professionals to increase efficacy and adherence to protective measures during pandemic.

## Introduction

In December 2019, China reported an emergence of a new coronavirus disease named COVID-19, caused by SARS-CoV-2. COVID-19 had spread rapidly across 220 countries worldwide. As of November 21, 2020, more than 58.2 million people have been infected with SARS-CoV-2 and over 1.3 million deaths have been documented globally [1]. In Morocco, the first cases have been diagnosed on March 2, 2020 [2]. As of November 21, 2020, a total of 320,962 cases have been diagnosed, with 5,256 corresponding deaths [2].

Healthcare workers, who are in frontline during outbreaks of infectious diseases related to airborne, are mandated to wear specific personal protective equipment (PPE) during their shifts for extended working hours. Since the start of the outbreak of COVID-19 in Morocco, healthcare workers in all hospitals and healthcare facilities have been asked to wear PPE including high-filtrating masks, goggles or visors additionally as protective jumpsuits or gowns and gloves while caring for suspected or confirmed COVID-19 patients.

The N95, the most used type of face mask so far, protects against respiratory droplets with the number 95 signifying that it is at least 95% efficient in filtering particles with a median diameter > 0.3 mm^2^, and the letter N that the mask is not resistant to oil [3].

Surgical or medical masks are loose-fitting, create a physical barrier, block larger particles, and are fluid-resistant. Cloth masks which are for non-medical use, vary regarding their filtration and fluid resistance depending on the material used, the number of layers, and fit. Single-use N95 and equivalent respirators provide higher respiratory protection than surgical masks and are required after an appropriate fitting before donning with other PPE [3-5].

Continuous use of PPE increases discomfort and exertion especially for extended hours of use [6]. Compressive headaches from the use of tight-fitting equipment have already been described in literature like helmet, swimming goggles and hat in other professions and activities [7-13]. During the COVID-19 pandemic and previous severe acute respiratory distress syndromes, De novo compressive headache has been described after the use of masks or a combination of masks and eye protections for long hours [14,15].

The aim of our study is to describe subtypes of headaches among frontline healthcare workers using PPE, during COVID-19, in Cheikh Khalifa International University Hospital of Casablanca (CKIUH), Morocco. Secondly, we sought to determine correlations between PPE related headaches among healthcare workers and their working conditions and trends in PPE use during pandemic.

## Materials and methods

Study design and setting

This is a cross-sectional study conducted at Cheikh Khalifa International University Hospital (CKIUH), a tertiary care university hospital situated in the Casablanca-Settat region, the largest one in the kingdom of Morocco with a population of 6,862,000 inhabitants and the most hit by COVID-19 pandemic in Morocco [16,17].

The hospital has organized its departments on “High-Risk areas” which included: emergency department with isolation and diagnosis area where patients were admitted for investigation; and in-ward hospitalization area with 42 single bedrooms for mild to moderate cases. Severe COVID-19 cases were managed at a dedicated critical care and intensive care units with a capacity of 23 beds. Additionally, a fully functional field hospital has been rapidly set up to receive more patients for isolation and follow-up [17].

Medical and paramedical staff were mandated to wear special personal protective equipment including masks all time, associated to goggles or protective glasses while doing procedures and caring for suspected or confirmed cases. Face shields have been also used during high-risk procedures.

Study population

The frontline healthcare workers of CKIUH meeting the following inclusion criteria were enrolled in this study. We included doctors, residents, interns, nurses and paramedics from all departments caring for suspected or confirmed COVID-19 patients. We excluded pregnant women or immune-deficient healthcare professionals not involved directly in the management of COVID-19 cases. 

Data collection

Data of this study were collected using an online questionnaire sent to all healthcare workers of our institution in frontline during the first hit of COVID-19 pandemic in April 2020.

The online questionnaire included six sections with the first section giving concise introduction of the study and a consent part that all participants approved before to start answering the questionnaire.

In section 1, we collected demographic data about age, gender, marital status and profession of respondents.

In section 2, we collected data about previous chronic or occasional headaches and its characteristics and any relevant comorbidities: hypertension, stroke, diabetes, cardiac or pulmonary diseases, sinusitis, rhinitis and also depression or anxiety. We also collected data about the consumption of coffee and toxicological habits: tobacco, alcohol and other toxics.

In section 3 of the online questionnaire, we were interested in describing work conditions of healthcare workers. We collected data about how many hours they were working per day, before and during the pandemic; the number of shifts per week they had, before and during the pandemic and also the number of hours they were working by shift. We also asked healthcare workers about their regular department and if they had to change departments during the COVID-19 pandemic.

In section 4, we asked the participants about the personal protective equipment they were using before versus during the pandemic: the type of facial masks and eye protection used and also the patterns and number of hours of use of these protective equipment.

In section 5, we collected data about headaches generated by wearing personal protective equipment and categorize them on “De Novo” headache (never experienced before) or aggravation of pre-existing headache, their intensity and time of onset and resolution when donning/doffing personal protective equipment. The localization of headache in terms of craniofacial areas and the need of pain medication were determined.

In section 6, we evaluated the overall impact of PPE use on the quality of life of healthcare workers. We asked the participants if they had difficulty breathing or experienced a blurred vision while wearing PPE and if they estimate that it’s impacting their concentration and work performance.

Statistical analysis

Data analysis was performed using SPSS (version 20) software (IBM Corp., Armonk, NY). The distributions of baseline characteristics were described as frequencies (percentages) for qualitative variables and means (± standard deviations) for continuous variables. The differences were assessed using chi-square test or Fisher’s exact test. P-values were two-tailed and considered statistically significant if lower than 0.05.

Ethical considerations

The study was approved by the Ethics Committee of the Mohammed VI University of Health Sciences (UM6SS) & Cheikh Khalifa International University Hospital (CHIUH). Information collected anonymously from participants was kept confidential. Electronic informed consent to participate in the study was obtained from all study participants.

## Results

A total of 155 healthcare workers responded to the questionnaire. The majority of respondents were females with 107 (69%) participants and 48 (31%) males. The mean age of participants was 32 (+/-9.32 SD) with a range from 22 to 64 years old. Of the respondents, the majority were doctors represented by 102 participants (65.8%) followed by nurses with 46 participants (29.7%) and 7 paramedics (4.5%). Table 1 summarizes demographic characteristics, comorbidities and toxic habits of the study population. The most prevalent comorbidities were rhinitis in 37 (23.9%) respondents, asthma in 19 (12.3%) respondents and depression or anxiety in 14 (9%) participants. Tobacco and other toxics use was reported by 14 respondents (9%). 81 (52.3%) respondents had previous chronic headache and migraine was the most prevalent type in 47 (30.3%) participants.

**Table 1:** Demographic characteristics and comorbidities among healthcare workers in our study SD: standard deviation; M: male; F: female

	Number (%); n = 155
Age (mean +/- SD)	32.03 +/- 9.3
Gender	
M	48 (31)
F	107 (69)
Marital status	
Single	82 (52.9)
Married	68 (43.9)
Divorced	5 (3.2)
Profession	
Doctors	102 (65.8)
Nurses	46 (29.7)
Paramedic	7 (4.5%)
Comorbidities	
Chronic or occasional headache	81 (52.3)
Migraine	47 (30.3)
Tension headache	28 (18.1)
Stroke	6 (3.9)
Hypertension	3 (1.9)
Insulin dependent Diabetes	1 (0.6)
Non-insulin dependent Diabetes	1 (0.6)
Sinusitis	6 (3.9)
Rhinitis	37 (23.9)
Cardiac disease	6 (3.9)
Asthma	19 (12.3)
Depression/ anxiety	14 (9)
Coffee consumption	77 (49.7%)
Toxicological habits	
Tobacco	14 (9)
Alcohol	2 (1.3)
Cannabis	2 (1.3)

During pandemic, healthcare workers were assessed to hospitalization wards (54.8%) followed by clinics (32.3%) and emergency departments (24.5%). Intensive care unit and critical care units represented 36.1% of assessed departments. 79 (51%) respondents were working 8 hours as regular working hours. Additionally, 98 (63.2%) were doing more than two shifts per week with 77 (49.7%) respondents working 8 to 12 hours per shift. Personal protective equipment use pattern by healthcare workers during the COVID-19 pandemic in our hospital is represented in Table 2. Over the 155 respondents, 148 healthcare workers (95.5%) were using N95 masks at the hospital during the pandemic and 61.3% of respondents were using eye protection either glasses (39.4%) and/or face-shield/visor (34.2%) and/or goggles (4.5%). They tend to use personal protective equipment for more than four hours either for masks (96.1%) or eye/face protection (67.1%).

**Table 2:** Personal protective equipment usage pattern during COVID-19 pandemic

	Number (%); n= 155
Type of masks	
Ffp3/Ffp2(N95)	148 (95.5)
Ffp1 or Surgical	7 (4.5)
Eyes Protective equipment	
No	60 (38.7)
Yes	95 (61.3)
Glasses	61 (39.4)
Goggles	7 (4.5)
Face shield	53 (34.2)
Number of hours wearing masks	
<4 hours	6 (3.9)
>4 hours	149 (96.1)
Number of hours wearing eyes protective equipment	
< 4 hours	51 (32.9)
> 4 hours	104 (67.1)

Table 3 shows the type and frequency of headaches generated by wearing PPE. 96 (62%) healthcare workers reported headache induced by the use of personal protective equipment during the pandemic. Forty-one (32.9%) respondents qualified this headache as De Novo headache, while 45 (29%) judged this headache as an already experienced headache but aggravated by the use of personal protective equipment. Headache induced by the use of PPE occurred less than twice a week for 60 patients (62.5%, n = 96) and more than two times a week for 36 patients (37.5%, n = 96).

**Table 3:** Type and frequency of headache generated by wearing PPE PPE: personal protective equipment

	Number, n = 155 (%)
PPE generated headache	
Yes	96 (62)
No	59 (38)
Type of PPE generated Headache	
De Novo	51 (32.9)
Aggravated chronic Headache	45 (29)
Frequency per weeks	
1 per 2 weeks	25 (26.05)
1 per week	35 (34.5)
2 per week	18 (18.7)
> 3 per week	18 (18.7)

**Figure 1:**
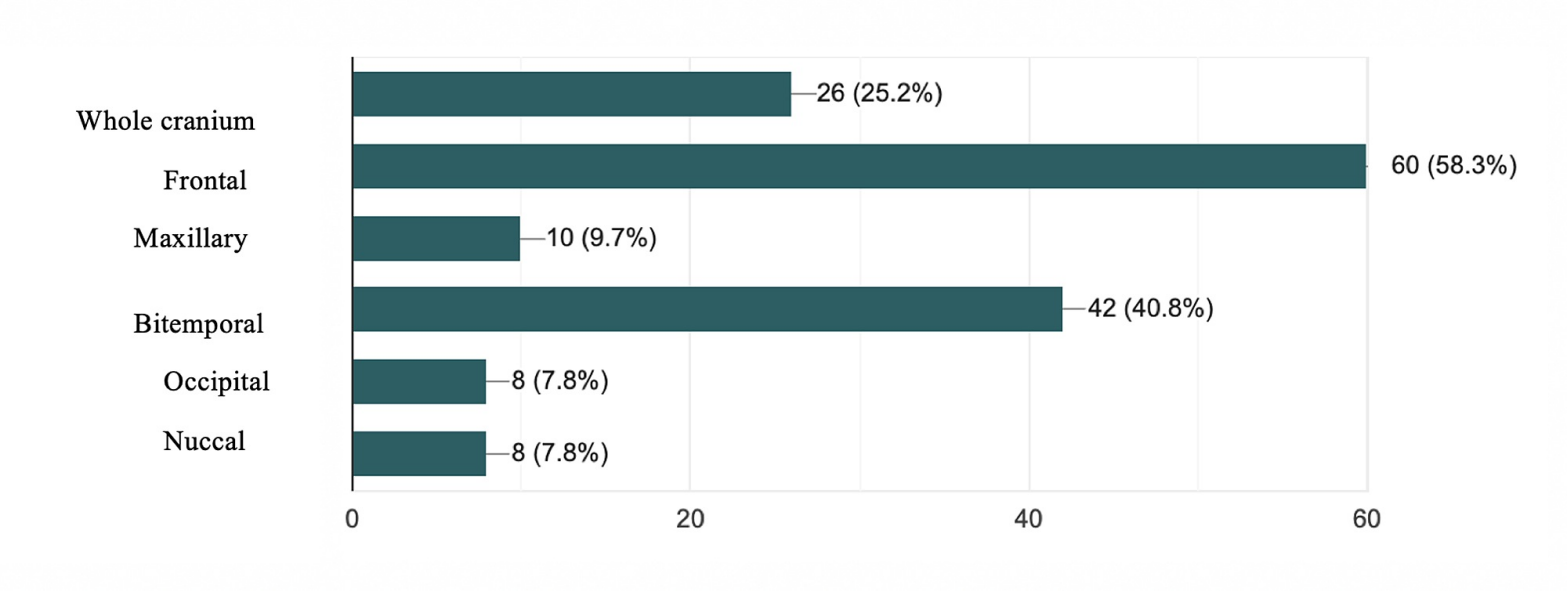
Localization of PPE generated headache in healthcare workers PPE: personal protective equipment

The headache onset after wearing masks was reported to be less than 60 min in 75% of respondents (n = 72 of 96). Also, the resolution of headache generated by masks was reported to be less than 60 min in 67.7% of respondents (n = 65 of 96). Pain medication was used by 90.62% of respondents (n = 87 of 96). The most used molecules were acetaminophen (55.2%), opioids (14.6%), non-steroidal anti-inflammatory (13.54%) while triptans were rarely used (5.2%). Figure 1 shows the predominant location of headache; frontal and bi-temporal areas are the most prevalent sites of headache generated by PPE use (58.3% and 40.8%, respectively). 

The study of correlation between demographic characteristics and De Novo headache showed no correlation between gender, age, marital status, profession, comorbidities, toxic habits and pre-existing headache with De Novo headache generated by PPE use (Table 4). Working more than eight hours per shift during pandemic was correlated to De novo generated headache (p = 0.008). However, the type of mask used by healthcare workers was not statistically correlated to De novo headache (p = 0.22) neither was the number of shifts per week (p = 0.6). All healthcare workers who reported De novo headache (n = 51) were wearing masks for more than four hours but the study of correlation doesn’t meet the statistical significance (p = 0.18). The number of hours wearing eye protection was also not correlated to De novo headache (p = 0.58).

**Table 4:** Study of correlation between demographic characteristics, working conditions, types of PPE used, duration of use and De Novo headache M: male; F: female; PPE: personal protective equipment. Toxic habits: refers to tobacco, cannabis and alcohol consumption.

	Without De Novo Headache, n = 104	With De NOVO Headache, n= 51	p-value
Gender			0.85
M	33	15	
F	71	36	
Age			0.73
< 30	53	28	
> 30	51	23	
Profession			0.37
Medical	71	31	
Paramedics	33	20	
Toxic habits			0.23
No	97	44	
Yes	7	7	
Coffee consumption			0.17
No	48	30	
Yes	56	21	
Previous Headache			0.39
No	47	27	
Yes	57	24	
Number of regular working hours			0.39
< 8h	56	23	
> 8h	48	28	
Number of shifts per week during pandemic			0.6
<2 per week	40	17	
> 2 per week	64	34	
Number of working hours per shift			0.008
< 8h	30	5	
> 8h	74	46	
Number of hours wearing Masks during pandemic			0.18
< 4 h	6	0	
> 4 h	98	51	
Number of Hours wearing eye protection during pandemic			0.58
< 4h	36	15	
> 4h	68	36	
Type of Mask used			0.22
FFP2, FFP3, N95	101	47	
Surgical masks	3	4	

For aggravated headache by wearing PPE, the study of correlation between demographic characteristics and this type of headache showed no statistical significant correlation for gender, age, toxic habits and coffee consumption (Table 5). However, the profession of doctor was correlated to PPE aggravated headache (p = 0.02). Working hours per shifts more than 12 hours was correlated to PPE aggravated headache (p = 0.023). However, wearing eye protection for more than four hours was not correlated to PPE aggravated headache (p = 0.06) neither was the frequency of shifts worked per week (p = 0.36). Although 44 over 45 respondents with PPE aggravated headache were wearing masks for more than four hours, no statistical correlation was found (p = 0.67). The type of mask used was not correlated to PPE aggravated headache occurrence in respondents (p =0.41).

**Table 5:** Study of correlation between demographic characteristics, working conditions, types of PPE used, duration of use and aggravated chronic headache by the use of PPE PPE: personal protective equipment

	Without Aggravated Headache, n= 110	With Aggravated Headache, n= 45	p-value
Gender			0.34
M	37	11	
F	73	34	
Age			0.72
< 30	56	25	
> 30	54	20	
Profession			0.02
Medical	66	36	
Paramedics	44	9	
Toxic habits			0.12
No	103	38	
Yes	7	7	
Coffee consumption			0.59
No	57	21	
Yes	53	24	
Previous Headache			0.00001
No	73	1	
Yes	37	44	
Number of regular Working Hours			1.0
< 8h	56	23	
> 8h	54	22	
Number of shifts per week during pandemic			0.36
<2 per week	43	14	
> 2 per week	67	31	
Number of working hours per shift			0.023
<12 h	82	25	
>12 h	28	20	
Number of hours wearing Masks during pandemic			0.67
< 4 h	5	1	
> 4 h	105	44	
Number of Hours wearing eye protection during pandemic			0.06
< 4h	31	20	
> 4h	79	25	
Type of Mask used			0.41
FFP2, FFP3, N95	106	42	
Surgical masks	4	3	

Healthcare workers reported an impact on work-related quality of life due to PPE use during the COVID-19 pandemic and experienced transient to permanent breathing discomfort in 72.7% (109 over 150) respondents. 58% of respondents reported blurred vision due to the use of eye protection and masks. Respondents reported no or mild reduction of their concentration while performing their work (64.5%) while participants judged their professional performance mildly reduced by the use of PPE in 66.9%.

## Discussion

In this study, we described demographic characteristics, comorbidities and work conditions of healthcare workers in our hospital during the first hit of COVID-19 pandemic in Morocco. Healthcare workers were using mainly specific high filtrating masks, the N95(ffp2) or ffp3 additionally to other personal protective equipment for eyes and face. They were wearing masks for a prolonged period of time (mask’s use for more than four hours in 96.1% of respondents and in 67.1% of respondents for eye protection). Work conditions had shifted to more prolonged working hours and shifts per week during the pandemic with mandatory use of specific personal protective equipment by standardized protocols during the pandemic. We described headaches associated with the use of personal protective equipment, either as “De Novo” when not experienced before or an “aggravated headache” in case of a pre-existing chronic headache that is intensified by the wear of PPE.

Our results show that 62% of healthcare workers in our institution suffered from headache associated with the use of PPE with 32.9% of de novo headaches and 29% of aggravated headaches. We found that working more than 8 hours per shift is correlated to the development of de novo headache in healthcare workers in frontline during COVID-19 pandemic; while working more than 12 hours per shift is correlated to an aggravation of pre-existing chronic headache in healthcare workers. Furthermore, being a doctor, having pre-existing chronic headache and wearing eye protection in association with masks for more than four hours are correlated to aggravation of chronic pre-existing headache in healthcare workers.

The majority of respondents reported the onset of headache around 60 minutes after wearing PPE and its resolution 60 minutes after removing it, and it was located mainly in frontal (58,3%) and temporal (40,8%) areas, which satisfied the definition of external compressive headache according to the International Classification of Headache Disorders, ICDH-3, 2018 [18]. However, other theories try to explain the pathogenesis of headache related to the use of masks and other PPE for an extended period of time and that besides mechanical factors, like hypoxia, hypercarbia and stress [19-21]. In a group of 10 ICU nurses who participated in the study of Rebmann et al., most nurses (90%) tolerated the use of N95 masks for two shifts of 12 hours. However, wearing an N95 for an entire 12-hour shift had statistically significant negative effects on some physiologic measures and subjective symptoms [19]. Over time, CO_2_ levels increased significantly compared with beginning-of-shift baseline measures. Perceived exertion, shortness of air and complaints of headache, light-headedness and difficulty communicating also increased [19]. There were no changes in blood pressure or Oxygen levels, perceived comfort, perceived thermal comfort, or complaints of visual difficulties compared with baseline levels [19]. Wearing N95 mask with surgical mask overlay increased significantly CO_2_ level, nausea and complaints of visual challenges while it doesn’t have a significant negative impact over wearing N95 alone on blood pressure, O2 levels, heart rate, headache, shortness of breath, perceived comfort and thermal comfort or impeded communication [19]. Interestingly, nurses with higher body mass index (BMI) had a significant negative effect on some physiological parameters than nurses with normal BMI [19]. Cerebral hemodynamic parameters are altered as well by the wear of N95 masks. Transcranial Doppler monitoring of the middle cerebral artery showed that the use of N95 masks induced a significant increase in mean flow velocity and pulsatility index. Moreover, Carbon dioxide end-tidal pressure increased [22]. Interestingly, the combined use of N95 mask with powered air-purifying respirator (PARP) was more comfortable compared to N95 mask alone. The additional use of PARP mitigated the effects described earlier [22]. 

In the study of Ong et al., over 158 healthcare workers in frontline during the COVID-19 pandemic in Singapore, there was a significant increase in the use of PPE. The prevalence of de novo headache was 81% when using N95 masks and protective eyewear in this series. The headache was bilateral in location with a time of onset and resolution after wearing/removing PPE less than 60 minutes which was in concordance with the compressive headache definition [14]. Furthermore, study participants with pre-existing primary headaches and who worked in emergency departments were more likely to develop De Novo PPE-associated headaches. Healthcare workers using PPE either N95 or Eye protective equipment or both for more than four hours and having pre-existing primary headache diagnosis have more chances to develop PPE-associated De novo headache. These characteristics were found to be independently associated to the development of de Novo headache. 91.3% of respondents in Ong et al study, with underlying pre-existing headache diagnosis, reported an aggravation of their background headache in terms of frequency and duration of attack which is in concordance with our finding as well [15].

In contrast with our study, we found less De Novo PPE induced headache. Ninety-six (62%) healthcare workers reported headache induced by the use of personal protective equipment. Fifty-one (32.9%) respondents qualified this headache as De Novo headache, while 45 (29%) judged this headache as an aggravation of their pre-existing headache. This is probably related to different working conditions and shift rotations per week in our hospital. In our study, some specific working conditions seemed related to the development or the aggravation of headache. Indeed, working more than eight hours per shift was related to De-Novo headache while working more than 12h hours per shift was related to the aggravation of pre-existing headaches in health professionals. 

During the 2003 severe acute respiratory distress syndrome epidemic (SARS), Lim et al. studied risk factors associated with headache during the use of N95 masks only without eye protection in healthcare workers. Only 37.3% of respondents reported headaches related to the N95mask. They found that pre-existing headache and wearing masks for more than four hours were associated to mask generated headache. The combination of other protective equipment to high filtrating masks seems to have an effect on generating headache and discomfort [15].

Shenal et al. studied discomfort (including headache and facial pressure) and exertion experienced by healthcare workers related to many respirators models and during eight hours shift with an interposed doffing period every two hours. Interestingly and not surprisingly discomfort but not exertion increased significantly with continuous use of respirator. The N95 had a significantly greater level than PARP at six and eight hours. N95 with exhalation valve seemed to be the most comfortable among respirators. The medical mask was mildly more comfortable but it’s not suitable for pandemic situations among healthcare workers in frontline. It prevents the spread of aerosolized contaminants from the wearer and does not protect the wearer from outside ones [6]. 

We found moderate breathing discomfort experienced by healthcare workers in our study. Healthcare workers judged their concentration mildly reduced, their vision mildly blurred and finally their concentration and technical performance mildly impacted. This impact on work-related quality of life, could be a limiting factor to professional’s adherence to the correct use of PPE. In a study of 19 volunteers randomly allocated to wear PPE suits in healthcare environments, Loibner et al. studied limiting factors for wearing PPE during six working hours the first day and four working hours the other day at different temperatures. The most prevalent limiting factors were reduced dexterity, impaired vision and back pain related to respirators of the fully ventilated suit. However, these factors had no negative impact on performance. Interestingly heat stress and liquid loss were limiting at an elevated working temperature of 28°C but not 22°C [23].

Our study and results have many limitations. Primarily, our findings should be generalized with precaution since it’s a monocentric study with a relatively small sample size, but we believe that our study population is quite representative of tertiary care and regional hospitals in Morocco involved in COVID-19 patient’s management since the beginning of the pandemic. Secondary, it’s an electronic questionnaire, some aspects of discomfort and their impact on patient care and technical tasks have not been studied deeply. Another aspect related to lack of food, water and sleep during the extended working hours could be an aggravating factor of headaches and should be evaluated in further studies.

## Conclusions

The increased use of PPE, especially high filtrating masks during COVID-19 outbreak is responsible for generating headaches in healthcare workers on frontline either De novo or as an aggravation of pre-existing one. Working conditions have a greater impact on generating these types of headaches more than any pre-existing health conditions or comorbidities. These findings should be considered to accommodate health professionals by reducing the number of working hours per shift and probably set up regular breaks to allow health professionals doffing masks and other PPE in a correct manner. These accommodations may reduce the occurrence of headaches and discomfort and rise up the adherence to PPE among healthcare workers, in times of pandemic where security and protection of healthcare workers is one of the biggest concerns of health authorities.
